# A Retrospective Analysis of the Importance of Biochemical and Hematological Parameters for Mortality Prediction in COVID-19 Cases

**DOI:** 10.7759/cureus.30129

**Published:** 2022-10-10

**Authors:** Sumanashree Mallappa, Arti Khatri, Gayatri BN, Padmaja Kulkarni

**Affiliations:** 1 Pathology, Kodagu Institute of Medical Sciences, Madikeri, IND; 2 Pathology, Chacha Nehru Bal Chikitsalaya, Delhi, IND

**Keywords:** c-reactive protein (crp), covid-19 retro, neutrophils, risk assessment, prognosis, biomarkers

## Abstract

Introduction

The recent coronavirus disease 2019 (COVID-19) pandemic has devastated the world’s health and economy and has devastatingly affected social and emotional spheres. Although it was the older population who faced the worst, a good number of the younger population also lost their lives. It was very important to predict who will progress to the worst clinical outcome. Consequently, quick and accurate ways of forecasting mortality in COVID-19 cases are essential to save lives. In this study, 11 predictive parameters, namely, D-dimer, creatinine, C-reactive protein (CRP), lactate dehydrogenase (LDH), ferritin, neutrophil/lymphocyte ratio (NLR), platelet/lymphocyte ratio (PLR), systemic inflammatory index (SII), platelet count, absolute neutrophil count (ANC), and absolute lymphocyte count (ALC), were studied for determining their significance as predictive parameters of mortality in COVID-19-affected patients.

Methods

We conducted a retrospective study of essential parameters, namely, D-dimer, creatinine, CRP, LDH, ferritin, NLR, PLR, SII, platelet count, ANC, and ALC, in confirmed COVID-19 cases for one year between 2020 and 2021. The medical information was obtained from the digital storage sources of the hospital. All cases were segregated into surviving and non-surviving cases. All parameters were collected, results were tabulated, and each individual parameter was then analyzed to see if it showed any significant deviations in non-surviving cases and could help predict mortalities. Statistical analysis was conducted using the latest version of the Statistical Package for the Social Sciences (SPSS) software (IBM SPSS Statistics, Armonk, NY, USA).

Results

Each of the parameters was individually studied. D-dimer, creatinine, LDH, ferritin, CRP, NLR, PLR, SII, and ANC showed a statistically significant increase in non-surviving cases. Compared to surviving cases, ALC and platelets showed a statistically significant decrease in non-surviving instances.

Conclusion

All the studied parameters showed significant deviations in non-surviving cases and could help predict mortalities. This study also stresses the utility of readily available hematological ratios such as NLR and SII for prognosis in COVID-19 subjects.

## Introduction

Although coronavirus has been a well-known pathological entity since older times, in the year 2019, a new strain of this virus emerged that caused a severe global health emergency, killing millions and damaging the salient resources of education, economy, health infrastructure, and mental peace of a majority of the world’s population [1]. Therefore, controlling the spread of infection and helping the infected to survive was a top priority for the entire world during the coronavirus disease 2019 (COVID-19) wave.

Severe acute respiratory syndrome coronavirus 2 (SARS-CoV-2) is a new type of coronavirus whose nucleic acid sequence is different from those of severe acute respiratory syndrome coronavirus (SARS-CoV) and Middle East respiratory syndrome coronavirus (MERS-CoV) [2,3]. SARS-CoV-2 infection is characterized by unregulated and uncontrolled activation of the innate immune response, which acti­vates the endothelium, complement, and he­mostatic system [4].

The most common symptoms of COVID-19 infection include fever, sore throat, cough, weariness, headache, and dyspnea. Other prevalent symptoms include sputum production, headache, hemoptysis, diarrhea, and lymphopenia [5-8]. However, some cases have also been observed to be asymptomatic [1,9]. Symptoms appear after an incubation period of approximately 5.2 days [10,11]. Reverse transcription-polymerase chain reaction (RT-PCR SARS-CoV-2) is a widely accepted test for reliable COVID-19 diagnosis. In addition, laboratory parameters such as various hematological counts, hematological ratios, and a few biochemical tests can also add significant value in deciding the disease severity and treatment [9].

A major concern in COVID-19 infections was that a group of cases (namely, the older population), cases with associated comorbidities, men as compared to women progress to severe infection and lose the battle of life [12]. Nearly 20% of patients with COVID-19 become critically ill, with mortalities ranging as high as 8.1%-33%. Hence, effective ways of predicting the subset of cases that progress to this group are the need of the hour [6,13,14].

The unchecked hyperimmune response was known to be the cause of mortality in many such cases, and this condition was manifested by alterations in various hematological and biochemical parameters. Some of these parameters have been studied in COVID-19 patients [15-24].

India saw the first COVID-19 wave in January 2020, which caused mortality mainly in older and comorbid populations, and by December, cases were very few. Again, in March 2021, India saw a significant increase in COVID-19 cases, causing the second wave. The second wave was more dangerous than the first wave, causing many casualties. By the end of 2021, the second wave had started declining. Recently, after May 2022, there is evidence of cases again increasing in a few places in India [25].

COVID-19 infection spreads from person to person via aerosols, fomites, and close contacts. The virus induces a systemic inflammatory response, leading to the activation of the immune response and the release of various mediators. The result is the alteration in the levels of different biochemical and hematological parameters [26].

With the rapid evolution of both the virus and the disease’s nature, it becomes crucial to extensively study these parameters to know at-risk cases and thereby reduce mortality. Also, the possibility of the world facing another wave of this pandemic in the near future cannot be ruled out.

With this background, we embarked on a study to know if any of the following parameters had a significant association with severe non-surviving COVID-19 infection cases: D-dimer, creatinine, C-reactive protein (CRP), lactate dehydrogenase (LDH), ferritin, neutrophil/lymphocyte ratio (NLR), platelet/lymphocyte ratio (PLR), systemic inflammatory index (SII), platelet count, absolute neutrophil count (ANC), and absolute lymphocyte count (ALC).

## Materials and methods

COVID-19 was manifested by alterations in various hematological and biochemical parameters. Some of these parameters have been studied in COVID-19 patients previously [15-24]. We conducted a retrospective study of 11 parameters, namely, D-dimer, creatinine, CRP, LDH, ferritin, NLR, PLR, SII, platelet count, ANC, and ALC, in confirmed COVID-19 cases in the COVID-19 hospital of Madikeri, India, for a duration of one year between 2020 and 2021. These parameters were selected as these are very important hematological and biochemical parameters, and some of them, especially ratios such as neutrophil/lymphocyte ratio (NLR), platelet/lymphocyte ratio (PLR), and systemic inflammatory index (SII), needed more in-depth research.

Ethical committee approval for the study was taken from the institutional ethical committee.

A total of 422 cases were included in this study, out of which 230 (54.5%) were COVID-19-positive surviving cases and 192 (45.5%) were COVID-19-positive non-surviving cases.

Information was collected from the digital storage sources of the hospital for confirmed COVID-19-positive cases. Data for each individual parameter were followed regularly, and the peak values were recorded for each patient. All COVID-19-positive patients were segregated into surviving and non-surviving cases. For each group, the results of all the parameters were collected, and hematological ratios, namely, NLR (ANC/ALC), PLR (platelet count/ALC), and SSI ((platelet × neutrophil)/ALC), were calculated for all cases. Results were tabulated and then analyzed using the latest Statistical Package for the Social Sciences (SPSS) software version (IBM SPSS Statistics, Armonk, NY, USA).

## Results

A total of 422 cases were included in this study, out of which 230 (54.5%) were COVID-19-positive surviving cases and 192 (45.5%) were COVID-19-positive non-surviving cases (Table 1).

**Table 1:** Distribution of cases based on their survival status

Variable	Category	Number	%
Survival status	Survivors	230	54.5%
	Non-survivors	192	45.5%

The age and gender demographics of the cases are tabulated in Tables 2, 3, 4.

**Table 2:** Age-wise distribution of cases

Variable	Category		Number	%
Age	≤20 years		21	5%
	21-40 years		89	21.1%
	41-60 years		139	32.9%
	61-80 years		160	37.9%
	>80 years		13	3.1%
	Total		422	100%
	Median	Range	Mean	Standard deviation
	55	3-94	53.63	18.53

**Table 3:** Gender-wise distribution of the study patients

Variable	Category	Number	%
Gender	Males	268	63.5%
	Females	154	36.5%

**Table 4:** Age and gender distribution between survivors and non-survivors *Statistically significant (P < 0.05)

Variable	Category	Survivors		Non-survivors		P value
		Mean	Standard deviation	Mean	Standard deviation	
Age	Mean and standard deviation	47.55	19.75	60.91	13.82	<0.001*
	Range	3-94		20-92		
Variable	Category	Number	%	Number	%	P value
Sex	Males	135	58.7%	133	69.3%	0.03*
	Females	95	41.3%	59	30.7%	

Age

Regarding age distribution, the maximum number of patients in our study was between the age group of 61 and 80 years with 160 (37.9%) cases. The mean age of the patients was 53.63 years, and the median age was 55 years. The mean age of survivors was 47.55 years and of non-survivors was 60.91 years (Tables 2, 4).

Gender

Out of the 422 cases, 268 (63.5%) cases were males and 154 (36.5%) cases were females. Among the survivors, 58.7% were males and 41.3% were females. Among the non-survivors, 69.3% were males and 30.7% were females (Tables 3, 4).

Comparison of mean values of different study parameters between survivors and non-survivors

A comparison of mean values of different study parameters between survivors and non-survivors using the Mann-Whitney test was made for D-dimer, creatinine, LDH, ferritin, CRP, NLR, PLR, SII, ANC, ALC, and platelet count. The results are tabulated in Table 5.

**Table 5:** Comparison of mean values of different study parameters between survivors and non-survivors using the Mann-Whitney test *Statistically significant (P < 0.05) LDH: lactate dehydrogenase, NLR: neutrophil/lymphocyte ratio, PLR: platelet/lymphocyte ratio, SII: systemic inflammatory index, ANC: absolute neutrophil count, ALC: absolute lymphocyte count, CRP: C-reactive protein

Parameters	Survivor			Non-survivor			P value
	Mean	Standard deviation	Median	Mean	Standard deviation	Median	
D-dimer	1,964.28	12,222.66	238.0	2,031.33	3,322.45	538.0	<0.001*
Creatinine	1.71	4.47	1.2	3.44	16.98	1.4	<0.001*
LDH	582.96	451.78	465.0	976.07	513.65	886.0	<0.001*
Ferritin	301.95	491.10	167.2	975.42	1,069.35	668.5	<0.001*
NLR	6.3	8.66	3.6	19.17	24.99	12.0	<0.001*
PLR	231.87	176.18	179.5	436.78	383.12	347.8	<0.001*
SII	13.85	19.40	7.7	38.64	59.89	21.5	<0.001*
ANC	5,202.17	2,806.18	4,500.0	7,755.26	3,992.26	6,850.0	<0.001*
ALC	1,343.91	732.13	1,300.0	744.27	620.65	500.0	<0.001*
Platelet	2.34	1.01	2.3	2.01	0.78	1.9	<0.001*
CRP	68.68	96.64	18.4	111.61	86.44	116.0	<0.001*

The mean values for D-dimer was 1,964.28 (survivors) and 2,031.33 (non-survivors) (P < 0.001), creatinine was 1.71 (survivors) and 3.44 (non-survivors) (P < 0.001), LDH was 5.82.96 (survivors) and 976.07 (non-survivors) (P < 0.001), ferritin was 301.95 (survivors) and 975.42 (non-survivors) (P < 0.001), CRP was 68.68 (survivors) and 111.61 (non-survivors) (P < 0.001), NLR was 6.3 (survivors) and 19.17 (non-survivors) (P < 0.001), PLR was 231.87 (survivors) and 436.78 (non-survivors) (P < 0.001), SII was 13.85 (survivors) and 38.64 (non-survivors) (P < 0.001), ANC was 5,202.17 (survivors) and 7,755.26 (non-survivors) (P < 0.001), ALC was 1,343.91 (survivors) and 744.27 (non-survivors) (P < 0.001), and platelet count was 2.34 (survivors) and 2.01 (non-survivors) (P < 0.001).

Raised D-dimer, creatinine, LDH, ferritin, CRP, NLR, PLR, SII, and ANC were statistically significant in non-surviving cases compared with surviving cases. Decreased levels of ALC and platelet counts showed a statistically significant association with non-surviving cases compared with surviving cases.

Receiver operating characteristic (ROC) curve analyses

**Figure 1:**
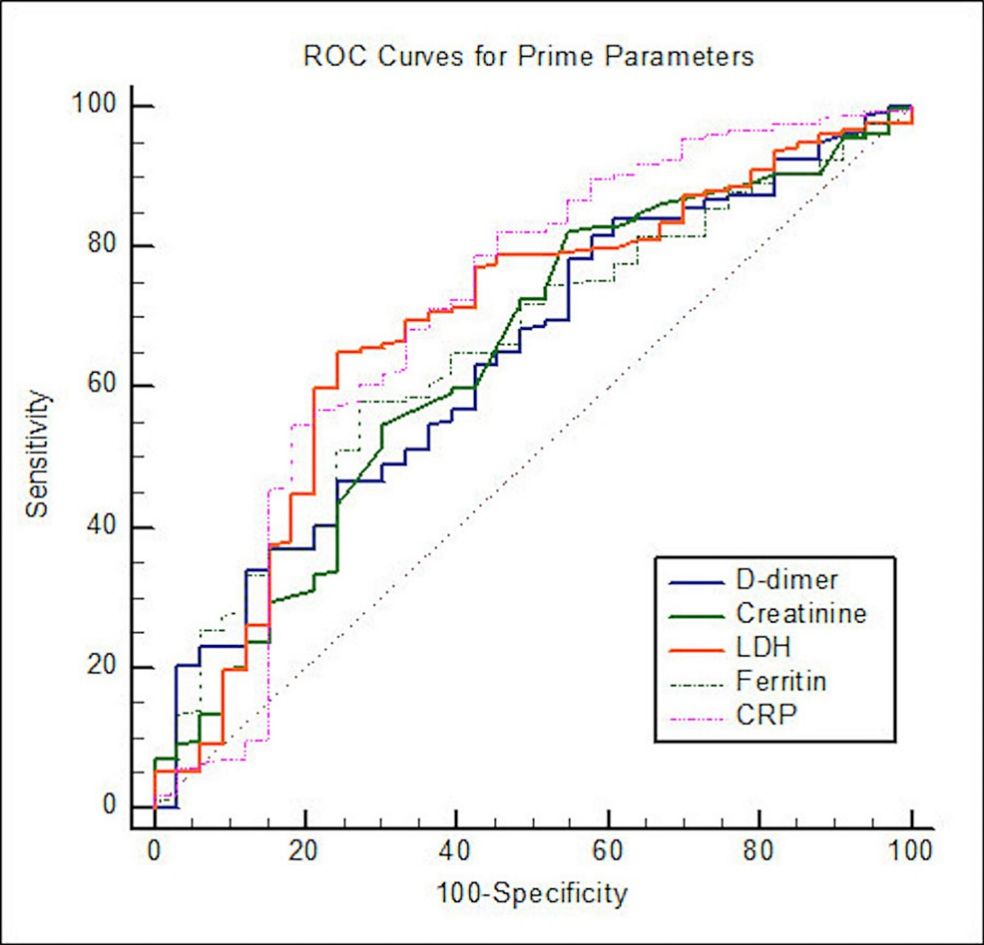
ROC curves for D-dimer, creatinine, LDH, CRP, and ferritin ROC: receiver operating characteristic, LDH: lactate dehydrogenase, CRP: C-reactive protein

**Figure 2:**
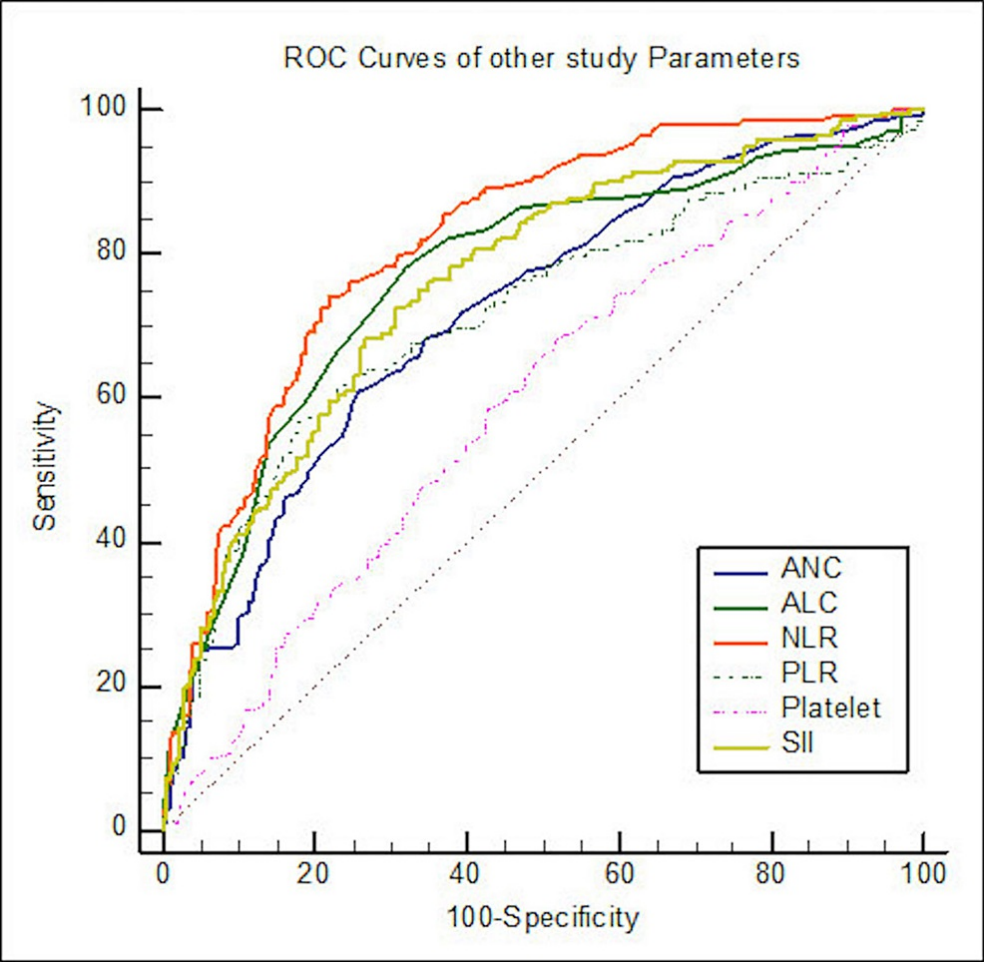
ROC curves for ANC, ALC, NLR, PLR, platelet count, and SII ROC: receiver operating characteristic, NLR: neutrophil/lymphocyte ratio, PLR: platelet lymphocyte ratio, SII: systemic inflammatory index, ANC: absolute neutrophil count, ALC: absolute lymphocyte count

ROC curve analyses for different study parameters for determining the cutoff between surviving and non-surviving patients were done with a 95% confidence interval (Table 6 and Figures 1, 2).

**Table 6:** ROC curve analysis for different study parameters for determining the cutoff between survivors and non-survivors *Statistically significant (P < 0.05) ROC: receiver operating characteristic, AUC: area under the curve, LDH: lactate dehydrogenase, NLR: neutrophil/lymphocyte ratio, PLR: platelet/lymphocyte ratio, SII: systemic inflammatory index, ANC: absolute neutrophil count, ALC: absolute lymphocyte count, CRP: C-reactive protein

Variable	AUC	Standard error	95% confidence interval		P value	Cutoff	Sensitivity (%)	Specificity (%)
			Lower	Upper				
D-dimer	0.64	0.05	0.57	0.71	<0.001*	>286	79.6	64.1
Creatinine	0.64	0.05	0.57	0.71	<0.001*	>1.3	56.2	74.2
LDH	0.69	0.05	0.62	0.75	<0.001*	>730	66.5	86.0
Ferritin	0.65	0.05	0.58	0.72	<0.001*	>431	64.4	80.1
CRP	0.71	0.06	0.64	0.77	<0.001*	>30.8	80.2	60.8
ANC	0.72	0.02	0.67	0.76	<0.001*	>6000	60.9	74.4
ALC	0.77	0.02	0.73	0.81	<0.001*	<900	78.1	67.8
NLR	0.82	0.02	0.78	0.85	<0.001*	>6.71	74.0	78.3
PLR	0.72	0.03	0.67	0.76	<0.001*	>312	56.8	82.2
Platelet	0.60	0.03	0.55	0.64	<0.001*	≤2.3	68.2	48.3
SII	0.76	0.02	0.72	0.80	<0.001*	>12.80	72.4	69.6

The area under the curve for D-dimer was 0.64 (P < 0.001), creatinine was 0.64 (P < 0.001), LDH was 0.69 (P < 0.001), ferritin was 0.65 (P < 0.001), CRP was 0.71 (P < 0.001), ANC was 0.72 (P < 0.001), ALC was 0.77 (P < 0.001), NLR was 0.82 (P < 0.001), PLR was 0.72 (P < 0.001), platelets was 0.60 (P < 0.001), and SII was 0.76 (P < 0.001).

## Discussion

COVID-19 crippled the world recently in many areas such as social, economical, health, and psychological spheres. Although many studies were done in this field, an in-depth study of various hematological and inflammatory parameters was needed to know if the deviations in any of these individual parameters were strongly associated with non-surviving cases as compared to surviving cases.

Although inflammation is an indispensable tool to fight viral infection, the overriding of the immune mechanisms can lead to disastrous consequences. The virus induces a systemic inflammatory response, leading to the activation of the immune response and the release of various mediators. The result is the alteration in the levels of different biochemical and hematological parameters [26]. The main issue with this pandemic is that a group of cases is progressing to severe disease and losing the battle of life, but finding out which set of cases will enter this group is the need of the hour [12].

These parameters have been used in risk assessment and to know if any of these predictive parameters can be significantly associated with mortality in these cases.

Seyit et al. [9] conducted a study where infected and non-infected groups were compared and concluded that CRP, LDH, NLR, PLR, and eosinophilia can serve as critical diagnostic tools. They also added that thrombocytopenia and PLR are also crucial in assessing the severity of the cases. Shang et al. [17] argued that NLR, CRP, and platelets could help in identifying the severity of the disease, and NLR was the best determinant of all three. Ding et al. [18] in their study found that leukocyte, neutrophil count, and neutrophil/lymphocyte ratio (NLR) were significantly higher in non-severe patients. In contrast, lymphocyte counts always tended to decrease in patients with severe disease. Qu et al. [19] said that the PLR is more closely associated with cytokine outbursts and can help in monitoring COVID-19 patients. Henry et al. [20] showed that LDH levels increased as the disease severity increased. Yang et al. [12] conducted a study where NLR was an independent prognostic biomarker that affected pneumonia progression in COVID-19 patients. NLR, LMR, PLR, CRP, and age were significantly higher in patients with severe disease. Liao et al. [15] showed that thrombocytopenia, increased NLR, increased prothrombin time, and increased D-dimer were associated with death. Zheng et al. [21] showed that neutrophil count, leucocyte count, and platelet count were independent risk factors for predicting the development of severe illness in COVID-19 cases.

In almost all these studies, few of the relevant parameters were included. However, with the rise of COVID-19 cases and the ever-changing knowledge of the virus, its natural history, and the role of various markers on severity, there is an immediate need for a deeper study. So, we embarked on a study to know the significance of the 11 parameters that were important in COVID-19 cases and compared these parameters and their behaviors in surviving and non-surviving COVID-19 patients.

A total of 422 cases were included in this study, of which 230 (54.5%) were COVID-19-positive surviving cases and 192 (45.5%) were COVID-19-positive non-surviving patients (Table 1).

Age distribution

The maximum number of patients in our study was between the age group of 61 and 80 years with 160 (37.9%) cases. The mean age of the patients was 53.63 years, and the median age was 55 years. The mean age of survivors was 47.55 years and of non-survivors was 60.91 years. The older the COVID-19 cases, the more the chances of them losing the battle of life. Increasing age was an independent statistically significant risk factor for mortality in these cases (Tables 2, 4).

Gender distribution

Out of the 422 cases, 268 (63.5%) cases were males and 154 (36.5%) cases were females. Among the survivors, 58.7% were males and 41.3% were females. Among the non-survivors, 69.3% were males and 30.7% were females. Males were more likely to progress toward a lethal fate when compared to females (Tables 3, 4).

Comparison of different study parameters between survivors and non-survivors

A comparison of mean values of varying study parameters between survivors and non-survivors using the Mann-Whitney test was done for D-dimer, creatinine, LDH, ferritin, CRP, NLR, PLR, SII, ANC, ALC, and platelets, and the results were tabulated (Table 5). D-dimer, creatinine, LDH, ferritin, CRP, NLR, PLR, SII, and ANC showed a statistically significant increase in non-surviving cases as compared with surviving cases. ALC and platelet count showed a statistically significant decrease in non-surviving cases as compared with surviving cases. The results of our study were found to closely align with the results of a few reported articles [9,15,17-20].

ROC curve analyses

ROC curve analyses for different study parameters for determining the cutoff between surviving and non-surviving patients were done with a 95% confidence interval. The area under the curve for D-dimer was 0.64 (P < 0.001), creatinine was 0.64 (P < 0.001), LDH was 0.69 (P < 0.001), ferritin was 0.65 (P < 0.001), CRP was 0.71 (P < 0.001), ANC was 0.72 (P < 0.001), ALC was 0.77 (P < 0.001), NLR was 0.82 (P <0.001), PLR was 0.72 (P < 0.001), platelets was 0.60 (P < 0.001), and SII was 0.76 (P < 0.001) (Table 6 and Figures 1, 2).

Although the rise of the five biochemical markers D-dimer, CRP, LDH, creatinine, and serum ferritin was significantly associated with mortality, CRP had the maximum area under the curve, and we wanted to know if any of these parameters were better than the other in predicting non-surviving cases. Therefore, a comparative ROC curve analysis was done. We found no statistically significant difference in the AUC among the five biochemical markers, and they were more or less equal in their predictive ability (Table 7).

**Table 7:** Comparison of ROC curve analysis of prime parameters to determine the prognostic importance in predicting the survival outcome ROC: receiver operating characteristic, AUC: area under the curve, LDH: lactate dehydrogenase, CRP: C-reactive protein, NB: null block (already compared parameters)

Variable	Parameters	Creatinine	LDH	Ferritin	CRP
AUC	D-dimer	0.00	0.05	0.01	0.06
	Creatinine	NB	0.05	0.01	0.06
	LDH	NB	NB	0.04	0.02
	Ferritin	NB	NB	NB	0.06

Similar comparative ROC curve analyses were done among the hematological ratio markers ANC, ALC, NLR, PLR, SII, and platelet count to know if any of them was better than the others in their severity-predicting ability. Platelet count had the least AUC among the ratios, and when compared with ANC, ALC, NLR, PLR, and SII ratios, the latter five were better predictors than platelet count (Table 8).

**Table 8:** Comparison of ROC curve analysis of prime parameters to determine the prognostic importance in predicting the survival outcome ROC: receiver operating characteristic, AUC: area under the curve, NLR: neutrophil/lymphocyte ratio, PLR: platelet/lymphocyte ratio, SII: systemic inflammatory index, ANC: absolute neutrophil count, ALC: absolute lymphocyte count, NB: null block (parameters already compared)

Variable	Parameters	ALC	NLR	PLR	Platelet	SII
Difference in AUC	ANC	0.05	0.10	0.00	0.12	0.04
	ALC	NB	0.05	0.05	0.17	0.01
	NLR	NB	NB	0.10	0.22	0.06
	PLR	NB	NB	NB	0.12	0.04
	Platelet	NB	NB	NB	NB	0.16

NLR had the maximum area under the curve (AUC) and was statistically better than ANC and PLR in predicting non-surviving cases. Among NLR, ALC, and ANC, NLR has greater severity prediction ability, followed by ALC, and then by ANC, as inferred by the area under the curve (AUC). However, all these parameters showed statistically significant alterations in non-surviving cases compared with surviving cases. NLR can be an important parameter in predicting fatality in COVID-19 infections. Yang et al. [12], Liao et al. [15], Shang al. [17], Ding et al. [18], Wang et al. [23], and Fu et al. [24] also in their respective studies said that NLR is a critical parameter that is raised in severe and fatal cases.

An article by Melenotte et al. [26] claims that in severe COVID-19 cases, marked myelopoiesis occurs, releasing immature neutrophils into the bloodstream. These immature neutrophils have immune-suppressive properties that can lead to immune thrombosis, sepsis deterioration, and infection spread. The findings of increased NLR in non-surviving cases of this study may have a similar underlying mechanism.

Moreover, neutrophils are triggered by various factors such as interleukin-6 and interleukin-8, tumor necrosis factor-alpha, granulocyte colony-stimulating factor, and interferon-gamma factors produced by lymphocyte and endothelial cells [27-30].

Systemic inflammatory index ((neutrophils × platelets)/ALC), a new parameter not much studied, was also included in our study and showed a statistically significant increase in fatal non-surviving COVID-19 cases when compared with surviving cases, with AUC of 0.76 (P < 0.001).

Ozdemir et al. [22] studied the ratios in COVID-19 cases and found that NLR and SII had the best predicting ability of fatality in COVID-19. Our study supports their claim.

Henry et al. [31] conducted a meta-analysis of numerous articles related to the topic. They wrote an article wherein the laboratory changes in patients with severe or fatal COVID-19 infection were summarized. Raised ANC, creatinine, LDH, D-dimer, CRP, serum ferritin, and platelet count along with decreased ALC and platelet count was associated with severe or fatal COVID-19 infections, and our results closely match their findings.

A study done by Lionte et al. [32] showed that these parameters and their correlation with mortality risk factors varied according to the variant of the virus infected. However, their study emphasized the importance of inflammatory parameters such as neutrophil/lymphocyte ratio (NLR), monocyte/lymphocyte ratio (MLR), C-reactive protein (CRP), and a few others and their importance in severe cases.

Overall, although all the parameters in our study proved statistically significant in assessing which cases can progress into fatal situations, losing the battle of life, we would like to highlight the importance of hematological ratios, which can be easily calculated at the bedside, with results that can be obtained fast. These ratios, especially NLR, can help immensely in emergencies with less time and resource-constrained diagnostic facilities. SII, a less studied ratio, also is significantly increased in non-surviving cases. All the biochemical parameters, especially CRP, can be helpful too.

Patient- and treatment-related inferences

The results of the study give important input on how these parameters behave with respect to survivor and non-survivor groups. It also tells their relative importance and stresses on less cumbersome and easily calculable ratios such as NLR, PLR, and SII, which can be easily calculated in less time even at the patient bedside and can be of immense help in monitoring prognosis as to which cases can progress to severity. Out of all the parameters, NLR showed the maximum AUC (0.82) and was statistically better than ANC (0.72) and PLR (0.72) in predicting non-surviving cases. Among NLR, ALC, and ANC, NLR (0.82) has greater severity prediction ability as inferred by the area under the curve (AUC), followed by ALC (0.77), and then by ANC (0.72).

The relative importance of each of these parameters is calculated based on the area under the curve (AUC) of each parameter, which is shown in the Results section. Out of all the parameters, NLR showed the maximum AUC (0.82), followed by ALC (0.77), SII (0.76), ANC (0.72), PLR (0.72), CRP (0.71), LDH (0.69), ferritin (0.65), D-dimer (0.64), creatinine (0.64), and platelets (0.60).

The limitation of this study was that it was a retrospective study.

## Conclusions

Although few studies have been done to study the predictive parameters of severity in COVID-19 cases, given the ever-evolving nature of the disease and the possibility of the world facing the resurgence of COVID-19 again in the future, there was a need for a more detailed study. The current study has 11 crucial parameters: D-dimer, creatinine, LDH, ferritin, CRP, NLR, PLR, SII, ANC, ALC, and platelets. ROC curve was plotted for each of these parameters, and the AUC were as follows: D-dimer, 0.64 (P < 0.001); creatinine, 0.64 (P < 0.001); LDH, 0.69 (P < 0.001); ferritin, 0.65 (P < 0.001); CRP, 0.71 (P < 0.001); ANC, 0.72 (P < 0.001); ALC, 0.77 (P < 0.001); NLR, 0.82 (P < 0.001); PLR, 0.72 (P < 0.001); platelets, 0.60 (P < 0.001); and SII, 0.76 (P < 0.001).

Raised levels of D-dimer, creatinine, LDH, ferritin, CRP, NLR, PLR, SII, and ANC and decreased platelet count along with ALC were statistically significant in non-surviving fatal cases of COVID-19 infections compared to surviving cases. Among them, NLR had the maximum area under the curve in ROC and proved to be the most critical predictive parameter. ALC and systemic inflammatory index can also be crucial predictive parameters and need to be studied further.

We highlight the predictive importance of hematological ratios, especially NLR, which are easy to calculate at the bedside and provide quick results. SII, a less studied ratio, also proved to be significantly increased in non-surviving cases, and all the 11 biochemical parameters studied in our study, especially CRP, are beneficial in assessing COVID-19 progression into mortality.
